# Preservation of the ovarian reserve and hemostasis during laparoscopic ovarian cystectomy by a hemostatic agent versus suturing for patients with ovarian endometriosis: study protocol for randomized controlled, non-inferiority trial (PRAHA-2 trial)

**DOI:** 10.1186/s13063-021-05431-1

**Published:** 2021-07-21

**Authors:** Hyunji Lim, Soo Jin Park, Haerin Paik, Jaehee Mun, Eun Ji Lee, Seungmee Lee, Whasun Lim, Gwonhwa Song, Seung-Hyuk Shim, Chae Hyeong Lee, Ga Won Yim, Hee Seung Kim, PRAHA Study Group

**Affiliations:** 1 Department of Obstetrics and Gynecology, Seoul National University Hospital, 101 Daehak-Ro, Jongno-Gu, Seoul, 03080 Republic of Korea; 2 Department of Obstetrics & Gynecology, Keimyung University School of Medicine, Daegu, 41931 Republic of Korea; 3 Department of Food and Nutrition, Kookmin University, Seoul, 02707 Republic of Korea; 4 Institute of Animal Molecular Biotechnology and Department of Biotechnology, College of Life Sciences and Biotechnology, Korea University, Seoul, 02841 Republic of Korea; 5 Department of Obstetrics and Gynecology, Konkuk University Medical center, Seoul, 05030 Republic of Korea; 6 Department of Obstetrics and Gynecology, Dongguk University Ilsan Hospital, Goyang, 10326 Republic of Korea

**Keywords:** Endometriosis, Laparoscopic ovarian cystectomy, Ovarian reserve, Hemostatic agent, Suturing

## Abstract

**Background:**

Endometriosis (EMS) can be implanted everywhere, especially in pelvic organs. EMS can be asymptomatic, but it can result in pelvic pain and infertility by inducing local inflammation and pelvic adhesion. The prevalence of EMS is about 10% in reproductive-age women and higher in women with pelvic pain or infertility. For young patients with ovarian EMS, laparoscopic ovarian cystectomy is effective in relieving pelvic pain and preventing local recurrence. However, there is a concern that the ovarian reserve would decrease after the operation because of the removal of a part of the normal ovarian tissue and thermal damage during hemostasis, which depends on the types of hemostasis such as bipolar electrocoagulation, suturing, and the use of a hemostatic agent. In this study, we aim to evaluate the protective effect for the ovarian reserve and hemostasis between a hemostatic agent and suturing during laparoscopic ovarian cystectomy for patients with ovarian EMS.

**Methods:**

This study is a randomized controlled, non-inferiority trial, where a total of 90 patients with ovarian EMS will be randomly assigned to the experimental (hemostatic agent) and control (suturing) groups. In the control group, a barbed suture will be applied for hemostasis, whereas a hemostatic agent will be applied in the experimental group. If two methods are insufficient, bipolar electrocoagulation will be applied for complete hemostasis. As the primary endpoint, the reduction rate of serum anti- Müllerian hormone (AMH) levels reflecting the ovarian reserve will be compared between the two groups 12 weeks after surgery. As secondary endpoints, we will compare the reduction rate of AMH level 48 weeks after surgery, the time required to complete hemostasis, the success rate of hemostasis within 10 min, and adverse events associated with operation.

**Discussion:**

We expect that the protective effect for the ovarian reserve and hemostasis may be comparable between the two methods, suggesting that a hemostatic agent may be preferred considering that it is easy to use during laparoscopic ovarian cystectomy.

**Trial registration:**

ClinicalTrials.govNCT04643106. Registered on 22 November 2020

**Supplementary Information:**

The online version contains supplementary material available at 10.1186/s13063-021-05431-1.

## Administrative information

**Table:** 

Title {1}	Preservation of the ovarian reserve and hemostasis during laparoscopic ovarian cystectomy by a hemostatic agent versus suturing for patients with ovarian endometriosis: study protocol for randomized controlled, non-inferiority trial (PRAHA-2 trial)
Trial registration {2a and 2b}.	ClinicalTrials.gov NCT04643106
Protocol version {3}	Version 1.1 18 June 2021
Funding {4}	PRAHA-2 trial is funded by MEDIFINE Corp Co. Ltd. and Dalim Medical Corp.
Author details {5a}	^1^Department of Obstetrics and Gynecology, Seoul National University Hospital, Seoul 03080, Republic of Korea. ^2^Department of Obstetrics & Gynecology, Keimyung University School of Medicine, Daegu 41931, Republic of Korea ^3^Department of Food and Nutrition, Kookmin University, Seoul 02707, Republic of Korea. ^4^Institute of Animal Molecular Biotechnology and Department of Biotechnology, College of Life Sciences and Biotechnology, Korea University, Seoul 02841, Republic of Korea ^5^Department of Obstetrics and Gynecology, Konkuk University Medical center, Seoul 05030, Republic of Korea ^6^Department of Obstetrics and Gynecology, Dongguk University Ilsan Hospital, Goyang 10326, Republic of Korea All authors are included in the PRAHA (PRservation of ovArian reserve and Hemostasis during ovArian cystectomy) study group.
Name and contact information for the trial sponsor {5b}	1) Kwon-Yong Lee; MEDIFINE Corp. Co. Ltd.; 25, Misagangbyeonseo-ro, Hanam-si, Gyeonggi-do, Republic of Korea; Tel : 02-3437-5412, Fax : 02-3437-5414, E-mail : contact@medifine.com 2) Hwan Gyu Jung; Dalim Medical Corp., 52-1, World Cup buk-ro, Mapo-gu, Seoul, Republic of Korea. Tel : 02-335-1656, Fax : 02-332-0628, E-mail: international@dalimpharm.co.kr
Role of sponsor {5c}	This study sponsors and funders had no role in study design; collection, management, analysis, and interpretation of data; writing of the report; and the decision to submit the report for publication.

## Introduction

### Background and rationale {6a}

Endometriosis (EMS) is a condition that endometrial tissues are present outside the uterus. The most common sites of EMS are pelvic organs and peritoneum, but it can sometimes be seen at distant sites. The prevalence of EMS is about 10% in reproductive women, and it is higher in women with dysmenorrhea or infertility (17–44%) [1]. Although some women with EMS have no symptoms, a significant number of women with EMS have several symptoms such as dysmenorrhea, dyspareunia, and chronic pelvic pain and suffer from relevant infertility and cancer because of local inflammation and adhesion by EMS in the pelvic cavity [2].

Up to now, diagnostic laparoscopy is the gold standard for histologic confirmation of EMS, but medical treatment can be considered when ovarian EMS and deep infiltrating nodules are identified on imaging studies such as ultrasonography and magnetic resonance imaging. The treatment of EMS is individualized in consideration of age, the severity of symptoms, birth plan, and so on. Initially, medical treatment using nonsteroidal anti-inflammatory drugs and hormonal agents such as oral contraceptives, progestins, and gonadotropin-releasing hormone agonists can be considered [3]. However, surgical resection of deep infiltrating nodules or ovarian cystectomy should be conducted to relieve symptoms and improve the pregnancy rate [4, 5].

Nevertheless, surgical techniques for ovarian cystectomy can affect the remaining ovarian reserve after surgery because damage to the normal ovarian tissues depends on the proficiency in surgery to leave the normal ovarian tissue as much as possible and the methods for bleeding control. Sometimes the serum level of anti-Müllerian hormone (AMH) is measured before and after surgery to estimate the ovarian reserve. In young women, especially subfertile women who want to have a baby, the serum AMH level is a valuable predictor of reproductive potential [6]. In terms of bleeding control during laparoscopic ovarian cystectomy, bipolar electrocoagulation is a traditional and easy method, but it is hard to avoid thermal damage to the normal ovarian tissue. On the other hand, suture of the ovarian tissue can induce hemostasis effectively while avoiding thermal damage. However, it can lead to ischemic damage to the ovarian tissue due to excessive suture [7].

Alternatively, recent studies have suggested that hemostasis with a hemostatic agent during laparoscopic ovarian cystectomy may be effective by showing that a hemostatic agent may be superior to bipolar electrocoagulation for protecting the ovarian reserve [8–12]. In the randomized controlled trial (RCT) of PReservation of the ovArian reserve and Hemostasis during laparoscopic ovArian cystectomy (PRAHA trial), the decline ratio of serum anti-Müllerian hormone (AMH) was greater after bipolar electrocoagulation than after the use of a hemostatic agent in patients with ovarian EMS (50.7% vs. 14.4%) despite no difference between the two methods in those with ovarian non-EMS, suggesting that a hemostatic agent instead of bipolar electrocoagulation during laparoscopic ovarian cystectomy should be considered to preserve the ovarian reserve in patients with ovarian EMS [13].

On the other hand, there is a lack of data for comparing the protective effect for the ovarian reserve between laparoscopic ovarian suturing and a hemostatic agent. Given that laparoscopic ovarian suturing may take a significant amount of time to get used to, the use of a hemostatic agent can be preferred for laparoscopic gynecologists if two methods have a similar effect to protect the ovarian reserve after laparoscopic ovarian cystectomy in patients with ovarian EMS. Thus, we designed this randomized controlled, non-inferiority trial of PReservation of the ovArian reserve, and Hemostasis during laparoscopic ovArian cystectomy by a hemostatic agent versus suturing for patients with ovarian EMS (PRAHA-2 trial), where we will compare the protective effect for the ovarian reserve and hemostasis between a hemostatic agent and laparoscopic ovarian suturing. If this study shows the similar efficacy between the two methods, we can expect that a hemostatic agent may be an alternative to suturing for protecting the ovarian reserve and hemostasis in women with ovarian EMS.

### Objectives {7}

This study aims to evaluate the protective effect for preserving the ovarian reserve and hemostasis between a hemostatic agent and suturing during laparoscopic ovarian cystectomy for patients with ovarian EMS. Thus, we will estimate the reduction rate of serum AMH levels for the ovarian reserve, the time required for complete hemostasis, andhemoglobin levels with estimated blood loss for hemostasis between the two methods.

### Trial design {8}

**Fig. 1:**
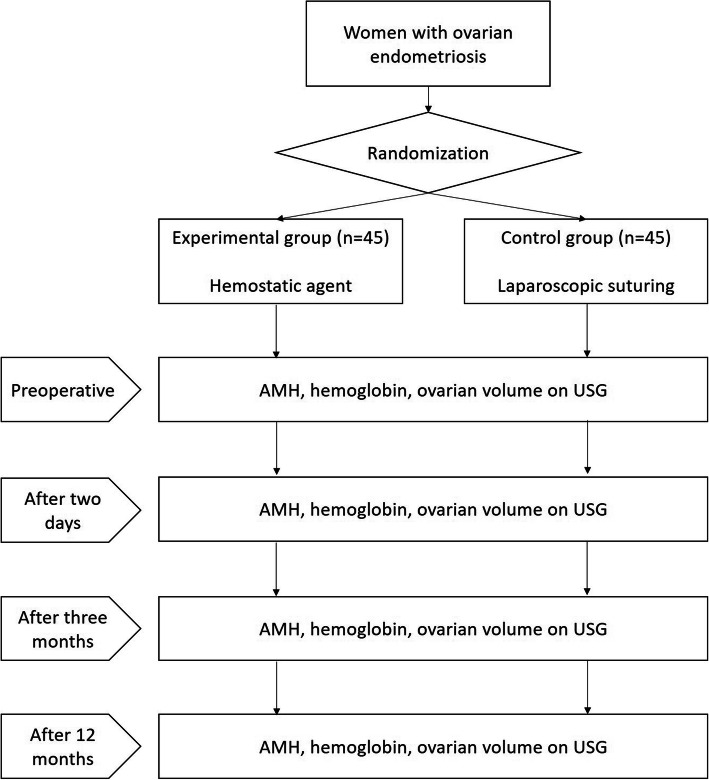
Schema of PRAHA-2 trial

This is an open-label, parallel-group, randomized controlled, non-inferiority trial. Participants with ovarian EMS will be randomly assigned to the experimental (hemostatic agent) and control (suturing) groups at a 1:1 ratio. After surgical treatment of ovarian EMS, we will check the serum AMH and hemoglobin levels and ovarian volumes measured by transvaginal or transrectal ultrasonography before surgery, after 2 days, after 3 months, and after 12 months. Figure 1 shows the schema of this study.

## Methods: participants, interventions, and outcomes

### Study setting {9}

As a multi-center trial, the study will be conducted at Seoul National University Hospital and Dongguk University Ilsan Hospital, in the Republic of Korea. All participants will be randomly assigned with a 1:1 ratio from the two hospitals.

### Eligibility criteria {10}

#### Inclusion criteria

The inclusion criteria are as follows:

##### Informed consent

1. Age 19–45-year-old women
2. American Society of Anesthesiologists Physical Status classification 1 or 2
3. Plan of laparoscopic ovarian cystectomy for unilateral or bilateral ovarian EMS diagnosed by imaging studies such as ultrasonography
4. 0.05 ng/ml or more of serum AMH levels
5. Regular menstruation every 21–45 days
6. Written and informed consent

#### Exclusion criteria

The exclusion criteria are as follows:

1. Non-EMS
2. Suspicious disease of ovarian malignancy
3. Age 18 and younger, 46 and older
4. Pregnancy or breastfeeding
5. Lower than 0.05 ng/ml of serum AMH levels
6. Hormonal therapy within recent 3 months
7. Considered as inappropriate by the researcher’s judgment
8. Refusal of written and informed consent

### Who will take informed consent? {26a}

Voluntary consent will be obtained in written form from all participants by the responsible party in this research: the research director or other relevant staff.

### Additional consent provisions for collection and use of participant data and biological specimens {26b}

There are provisions for the collection and use of participant data in the relevant study in the written consent.

### Interventions

#### Explanation for the choice of comparators {6b}

In laparoscopic ovarian cystectomy, not only to control bleeding, but it is also important to preserve the ovarian reserve. There are several methods to control bleeding during the operation, such as bipolar electrocoagulation, suturing, and the use of a hemostatic sealant. However, there are insufficient data to compare the effect to preserve the ovarian reserve and hemostasis between a hemostatic agent and laparoscopic suturing for patients with ovarian EMS. Therefore, this study is designed to show the similar effect of a hemostatic agent compared to laparoscopic suturing for the preservation of the ovarian reserve and hemostasis during laparoscopic ovarian cystectomy.

### Intervention description {11a}

#### Control group (suturing group)

During operation, a barbed suture will be applied to the inner surface of the ovarian parenchyme where ovarian EMS was attached.

#### Experimental group (hemostatic agent group)

During laparoscopic ovarian cystectomy, bleeding will be controlled by using a hemostatic agent (EVICEL®, Johnson and Johnson, Republic of Korea), which will be supported by Dalim Medical Corp. (Seoul, Republic of Korea).

### Criteria for discontinuing or modifying allocated interventions {11b}

If the designated methods for bleeding control are not sufficient, additional electrocoagulation using the energy device (POWERBLADE, MEDIFINE Corp. Co. Ltd., Republic of Korea) will be conducted without modification of allocation.

### Strategies to improve adherence to interventions {11c}

This item is not applicable because the intervention is conducted by a member of research investigators.

### Relevant concomitant care permitted or prohibited during the trial {11d}

The major outcome measures are the decline ratio of serum AMH levels after operation. So that taking medicine that can affect the level of AMH, such as hormonal drugs, must be carefully monitored. The patient who had taken any hormonal medication within 3 months before the operation should be excluded. However, after the operation, some people need to continue additional medical therapy to prevent the recurrence of EMS. So, it is permitted to take any hormonal medication after surgery under conditions that are completely monitored by research investigators.

### Provisions for post-trial care {30}

There is no post-trial care scheduled.

### Outcomes {12}

#### Primary outcome

1. The decline ratio of serum AMH levels 12 weeks after surgery: the decline ratio of serum AMH levels is defined as the value of (preoperative AMH levels − postoperative AMH levels)/(preoperative AMH levels). Serum AMH levels will be quantitatively measured by using an enzyme-linked immunosorbent assay.

#### Secondary outcome

1. The decline ratio of serum AMH levels 48 weeks after surgery.
2. The time required for complete hemostasis: stop-watch will be operated right after finishing to remove EMS tissues. It will be measured how long the time has been spent on bleeding control.
3. Success rates of hemostasis within 10 min: it will be evaluated whether hemostasis is finished within 10 min.
4. Blood loss during operation: the volume of blood loss will be estimated by using a simple visual assessment technique referring to gauze count and irrigation bottle.
5. Hemoglobin: 2 days, 12 weeks, and 48 weeks after surgery.
6. Transfusion during admission.
7. Adverse events associated with the operation, bleeding, or transfusion.
8. Hospitalization (days).
9. Operation time (min).

### Participant timeline {13}

All patients with EMS will be screened with history taking, laboratory tests, physical examinations, and ultrasonography. After admission for operation, written and informed consent will be obtained from patients who agree to enroll in the trial. Then, the subjects will be randomly assigned to one of the two groups. All patients will get routine hospital care with additional tests of serum hemoglobin and AMH levels and transvaginal/transrectal ultrasonography 2 days after surgery. These tests will be repeated at 3 months and 12 months after surgery. Supplementary Table 1 shows the schedule of enrollment, interventions, and assessments for participants.

### Sample size {14}

The number of participants is determined on the basis of the decline ratio of serum AMH levels 3 months after surgery with a significance level of 2.5% and the test power of 80%. For patients with ovarian EMS treated with laparoscopic suturing, the decline ratio of serum AMH levels 3 months after surgery has been reported to be 24.6% (interquartile range [IQR], 11.6–37.0) in the previous study [14]. From IQR and with the assumption of a normal distribution, standard deviation (SD) was calculated to be 18.81, where SD = IQR/1.349. Although the information on confidence intervals from relevant trials is required for determining the non-inferior margin, there appears no available study for confidence intervals. In one study on the comparison of the preservation of ovarian reserve between hemostatic suturing and electrocoagulation, it was reported that there was no significant difference between the two groups with respect to a reduction rate of serum AMH 3 months after surgery (suturing group 44% ± 28% vs. electrocauterization group 58% ± 24%, *p* = 0.15) [15]. Based on the 14% difference in the rate, we have calculated appropriate numbers of participants for the non-inferior margin from 8 to 14%. The smaller the non-inferior margin, the larger the sample size. Therefore, we set the non-inferior margin at 12% for the primary outcome, which will not be applied for the secondary outcomes because of a lack of relevant references. For the statistical test on non-inferiority with a mean difference of 0, the value for the sample size (*n* = 80) is available with SD of 18.81, and a non-inferior margin of 12% between the two groups, suggesting that the number of participants of each group is estimated to be 40. Supposed that the dropout rate is 10%, the number of participants should be 90 (45 per group).

### Recruitment {15}

We will enroll patients with unilateral or bilateral ovarian EMS who are to undergo laparoscopic ovarian cystectomy at Seoul National University Hospital and Dongguk University Hospital in the Republic of Korea. For achieving adequate participant enrollment to reach the target sample size, more institutions will be considered to be included during this trial.

### Assignment of interventions: allocation

#### Sequence generation {16a}

A randomization table will be made by using a reproducible website program (http://randomization.com). The table will be managed by one staff who is a gynecologist but not associated with this trial.

#### Concealment mechanism {16b}

Eligible patients will be allocated to receive the designated intervention during operation. However, she will get to know her allocation after the operation. On the other hand, the operator will be unblinded just before surgery to conduct the appropriate intervention for the patient. Actually, this current study is open-labeled.

#### Implementation {16c}

One designated staff will generate the allocation sequence by using the randomization program. After research investigators get informed consent from the subject, the staff will assign the patient to the determined group. The operator and other related investigators will be unblinded just before surgery to perform proper management.

### Assignment of interventions: blinding

#### Who will be blinded {17a}

This item is not applicable because the current research is open-labeled.

#### Procedure for unblinding if needed {17b}

This item is not applicable because the current research is open-labeled.

### Data collection and management

#### Plans for assessment and collection of outcomes {18a}

Research investigators are responsible for collecting the baseline, outcome, and other trial data. All data collected will be double-checked by the research staff. All associated assessors will be educated for subjective items: how to measure the ovarian volume by ultrasonography and time to spend to control bleeding, how to estimate the volume of blood loss during operation, and so on.

#### Plans to promote participant retention and complete follow-up {18b}

For all participants, the pelvic examination by ultrasonography will be provided twice (12 weeks and 48 weeks after surgery) for free of charge. If the patient wants to withdraw from this study, she can do it at any time. Investigators must describe the withdrawal reason in the electronic case report form. Investigators may request the patient to use data already collected before the withdrawal. If one agrees to that, incomplete data will be included for the assessment of the results.

#### Data management {19}

As soon as collected, all data will be typed in the electronic case report form by the data manager. Each completed electronic case report form will be double-checked by one or more research investigators. The data without any personal identification information are securely stored in the database. The file of eCRF is locked with a password and accessible to only the designated data manager. The ethics committee of Seoul National University Hospital and Dongguk University Ilsan Hospital will audit study conduct per 12 months.

#### Confidentiality {27}

After data collection, all personal identification information will be deleted, and sequential numbers will be given to data as study subject ID.

#### Plans for collection, laboratory evaluation, and storage of biological specimens for genetic or molecular analysis in this trial/future use {33}

The initial laboratory test, including complete blood count, liver enzyme, BUN, creatinine, and electrolyte will be conducted before operation. After each intervention, only complete blood count will be measured three times: 2 days, 12 weeks, and 48 weeks after the operation. There are no plans to use blood samples in the future.

## Statistical methods

### Statistical methods for primary and secondary outcomes {20a}

Both per-protocol (PP) and intention-to-treat (ITT) populations will be analyzed in two groups. The result will be regarded as valid when the experimental group is not inferior to the control group for the primary outcome, preservation of the ovarian reserve, in both analyzing methods. Serum AMH levels will be analyzed by using the Student *t* or Mann-Whitney *U* tests and repeated measure ANOVA. For secondary endpoints, variables will be analyzed by using the chi-square or Fisher’s exact test for categorical data and the Student *t* or Mann-Whitney *U* test for numerical data. *p* value < 0.05 is considered statistically significant.

### Interim analyses {21b}

There is no plan of any interim analyses to evaluate the efficacy or worthlessness of this trial. Because both interventions are already widely conducted during laparoscopic ovarian cystectomy, regardless of the current study, there is no reason to terminate the trial even if the results are not statistically significant

### Methods for additional analyses (e.g., subgroup analyses) {20b}

A separate supporting analysis will be conducted for unilateral or bilateral ovarian EMS.

### Methods in analysis to handle protocol non-adherence and any statistical methods to handle missing data {20c}

Both per-protocol (PP) and intention-to-treat (ITT) populations will be analyzed in the two groups. The results will be valid when it is accordant in both analyzing methods.

### Plans to give access to the full protocol, participant-level data, and statistical code {31c}

There are no detailed plans for this item.

## Oversight and monitoring

### Composition of the coordinating center and trial steering committee {5d}

#### Coordinating center

MRCC will act as the coordinating center for this trial.

#### Trial steering committee

Hyunji Lim, Soo Jin Park, Jaehee Mun, Haerin Paik, Eun Ji Lee, and Hee Seung Kim (Seoul National University Hospital, Republic of Korea).

Ga Won Yim and Chae Hyeong Lee (Dongguk University Ilsan Hospital).

### Composition of the data monitoring committee, its role, and reporting structure {21a}

Seungmee Lee (member): a professor at the Department of Obstetrics & Gynecology in Keimyung University School of Medicine, Daegu, Republic of Korea

Whasun Lim (member): a professor at the Department of Food and Nutrition in Kookmin University, Seoul, Republic of Korea

Gwonhwa Song (member): a professor at the Institute of Animal Molecular Biotechnology and Department of Biotechnology, College of Life Sciences and Biotechnology

Seung-Hyuk Shim (chairman): a professor at the Department of Obstetrics and Gynecology, Konkuk University Medical Center, Seoul, Republic of Korea

The data monitoring committee (DMC) consists of the four basic and clinical professors. All members are independent of the sponsor and competing interests. Designated investigators send a report, including registration of participants, intervention allocation, reasons for withdrawal, adverse event, and violation of initial protocol, to the DMC members 2 weeks before the DMC meeting. The meetings are scheduled to take place every 6 months but can be held more frequently if concerns arise. Any recommendations of the DMC are immediately passed on to the principal investigator.

### Adverse event reporting and harms {22}

Adverse events will be collected from the time of intervention to 12 months after the operation. As soon as recognizing the event, relevant investigators will fill them in the electrical case report form in detail. The principal investigator should inform the medical research ethics review committee of this event within 15 days. However, in the current study, interventions conducted in both groups are already done widely during laparoscopic ovarian cystectomy, so that it is expected that there are no additional harms derived from enrollment of this trial. Therefore, all participants get routine hospital care without any specific ancillary and post-trial care.

### Frequency and plans for auditing trial conduct {23}

The principal investigator will submit an interim report per 12 months, and the auditing is annually conducted by designated members of the Medical Research Collaborating Center (MRCC) from Seoul National University Hospital. The auditing process will be independent of investigators and the sponsor.

### Plans for communicating important protocol amendments to relevant parties (e.g., trial participants, ethical committees) {25}

During the current research, the principal investigator should inform all co-investigators from the participating hospitals of the revised protocol with accuracy. Then, they should inform the institutional review board (IRB) of their hospitals of any important protocol modifications. The modified protocol will only be implemented after receiving IRB approval in each relevant hospital. Also, the revised consent should be got in written form for all participants.

### Dissemination plans {31a}

The results of this trial will be shared with participants, healthcare professionals, the public, and other relevant groups via publication or presentation.

## Discussion

Up to now, relevant RCTs have shown no difference in the hemostatic effect between bipolar electrocoagulation and the use of a hemostatic agent [12, 14, 16–18]. This result means that the use of a hemostatic agent can reduce the frequency of use of bipolar electrocoagulation, thereby minimizing thermal damage to the ovarian tissues. In general, the ovarian reserve is defined as the number and quality of the ovarian follicles, and serum AMH levels are known to reflect the ovarian reserve well [9, 19]. Thus, some RCTs have suggested that the use of a hemostatic agent may be more beneficial for preserving the remaining ovarian reserve after laparoscopic ovarian cystectomy by showing that the decline ratio of serum AMH levels was lower in patients treated with a hemostatic agent than in those treated with bipolar electrocoagulation [12, 14, 17].

However, the PRAHA trial showed that the protective effect of a hemostatic agent was observed in only patients with ovarian EMS with no difference in the decline ratio of AMH between the two treatments in those with ovarian non-EMS. It means that the effort to minimize the removal of healthy ovarian tissue and the use of bipolar electrocoagulation can be helpful in preserving the ovarian reserve after laparoscopic ovarian cystectomy in most patients with ovarian cysts. However, we commonly conduct adhesiolysis sufficiently from tissues surrounding the lesion for patients with ovarian EMS. During the procedure, the vascular system within the ovarian cortex or surrounding the ovary can be injured, which can lead to lower serum AMH levels by an inadequate blood supply in the patients [20, 21].

Based on these results of the PRAHA trial, we aimed to enroll only patients with ovarian EMS for comparing the protective effect for the ovarian reserve and hemostasis between laparoscopic suturing and the use of a hemostatic agent in the PRAHA-2 trial. Although laparoscopic suturing can result in mechanical damage to the normal ovarian tissue and increased intra-ovarian pressure in ischemic regions, relevant trials comparing the ovarian reserve and hemostasis after laparoscopic ovarian cystectomy between suturing and bipolar electrocoagulation have reported that bipolar electrocoagulation may have a similar effect for hemostasis to suturing, but further reduce the ovarian reserve [13, 22]. When we consume that the hemostatic effect may be similar between suturing and a hemostatic agent, we can expect that suturing can be less beneficial than the use of a hemostatic agent due to the potential of ischemic damage to the ovarian tissue after suturing. Nevertheless, a recent systematic review suggested that suturing for hemostasis may be recommended compared to bipolar electrocoagulation and the use of a hemostatic agent based on the results of previous trials, suggesting that additional hemostasis using bipolar electrocoagulation may be more required during the use of a hemostatic agent than during suture [23]. Most importantly, there is no well-designed trial for comparing the protective effect for the ovarian reserve and hemostasis between suturing and the use of a hemostatic agent for patients with ovarian EMS. Since we estimated the sample size as logically as possible based on the existing research results, we believe that the PRAHA-2 trial will show the definite comparative results between laparoscopic suturing and a hemostatic agent, which will be helpful for preserving the ovarian reserve with effective hemostasis during laparoscopic ovarian cystectomy for patients with ovarian EMS.

## Trial status

The protocol version is number 1.0, dated 22 November 2020. We have not recruited the first patient, who is anticipated to be enrolled in January 2021.

## Supplementary Information

**Additional file 1: Table S1.** Schedule of enrolment, interventions, and assessments. Abbreviation: AMH, anti-Müllerian hormone. *Initial laboratory test includes complete blood count, liver enzyme, BUN, creatinine, electrolyte, etc. After operation, only complete blood count will be measured on every visit. **Ovarian volume is measured by transvaginal or transrectal ultrasonography.

## Data Availability

The data generated during the current study will be made available.

## Abbreviations

EMS: Endometriosis
RCT: Randomized controlled, non-inferiority trial
