# Supplementary material for: Preservation of the ovarian reserve and hemostasis during laparoscopic ovarian cystectomy by a hemostatic agent versus suturing for patients with ovarian endometriosis: study protocol for randomized controlled, non-inferiority trial (PRAHA-2 trial)
Source: Trials. 2021 Jul 21;22:473. doi: 10.1186/s13063-021-05431-1 (PMC8293561; doi:10.1186/s13063-021-05431-1)
Supplement: Supplementary file 1 — Additional file 1: Table S1. Schedule of enrolment, interventions, and assessments. Abbreviation: AMH, anti-Müllerian hormone. *Initial laboratory test includes complete blood count, liver enzyme, BUN, creatinine, electrolyte, etc. After operation, only complete blood count will be measured on every visit. **Ovarian volume is measured by transvaginal or transrectal ultrasonography. [file 13063_2021_5431_MOESM1_ESM.docx]

Supplementary table 1. Schedule of enrolment, interventions, and assessments

| **PRAHA-2 Trial** | **STUDY PERIOD** | | | | | |
| --- | --- | --- | --- | --- | --- | --- |
|  | **Enrolment** | **Allocation** | **Post-allocation** | | | |
| **TIMEPOINT** | ***Within 4 weeks*** | **D0** | ***D2*** | ***Week 1*** | ***Month 3*** | ***Month 12*** |
| **ENROLMENT:** |  | | | | | |
| **Eligibility screening** | X |  |  |  |  |  |
| **Informed consent** | X |  |  |  |  |  |
| **Premedical and operation history** | X |  |  |  |  |  |
| **Randomization** |  | X |  |  |  |  |
| **Allocation** |  | X |  |  |  |  |
| **INTERVENTIONS:** |  | | | | | |
| **Hemostatic agent application** |  | X |  |  |  |  |
| **Laparoscopic suturing** |  | X |  |  |  |  |
| **ASSESSMENTS:** |  | | | | | |
| **Medication** | X | X | X | X | X | X |
| **Vital sign** | X | X | X | X | X | X |
| **Laboratory test*** | X |  | X |  | X | X |
| **Serum AMH** | X |  | X |  | X | X |
| **Physical examination** | X | X | X | X | X | X |
| **Surgical risk**  **factors** |  | X | X | X |  |  |
| **Adverse event** |  | X | X | X |  |  |
| **Pelvic exam and ultrasonography**** | X | X | X |  | X | X |

Abbreviation: AMH, anti-Müllerian hormone

* Initial laboratory test includes complete blood count, liver enzyme, BUN, creatinine, electrolyte, etc. After operation, only complete blood count will be measured on every visit.

** Ovarian volume is measured by transvaginal or transrectal ultrasonography.
